# Supplementary material for: Fertilization and seasonality influence on the photochemical performance of tree legumes in forest plantation for area recovery in the Amazon
Source: PLoS One. 2021 May 21;16(5):e0243118. doi: 10.1371/journal.pone.0243118 (PMC8139463; doi:10.1371/journal.pone.0243118)
Supplement: S1 Table — F0, initial fluorescence; FM, maximum fluorescence; FV, maximum variable fluorescence; FV/FM, maximum quantum yield of PSII photochemistry; PIABS, performance index on an absorption basis; ET0/TR0, efficiency of electron transport; DI0/RC, energy dissipation flux; ABS/RC, antenna size of an active PSII RC; ET0/RC, electron transport flux; TR0/RC, maximum trapped exciton flux; RC/CS, density of reaction centers per cross-section. (DOCX) [file pone.0243118.s001.docx]

|  | *Cenostigma tocantinum* | *Senna reticulata* | *Dipteryx odorata* | *Clitoria fairchildiana* | *Inga edulis* | *Acacia spp.* |
| --- | --- | --- | --- | --- | --- | --- |
| *Dry season and low-nutrient* | | | | | | |
| *F_0_* | 561.63 ± 92.5 | 674.13 ± 87.2 | 716.94 ± 146.8 | 716.93 ± 103.0 | 660.75 ± 173.4 | 437.21 ± 40.7 |
| *F_M_* | 1931.81 ± 348.7 | 2210.50 ± 133.4 | 1661.31 ± 412.9 | 2431.86 ± 143.7 | 1953.13 ± 308.0 | 1788.64 ± 252.9 |
| *F_V_* | 1370.19 ± 329.0 | 1536.38 ± 144.6 | 944.38 ± 436.3 | 1714.93 ± 206.0 | 1292.38 ± 186.1 | 1351.43 ± 240.2 |
| *F_V_/F_M_* | 0.70 ± 0.1 | 0.69 ± 0.0 | 0.54 ± 0.1 | 0.70 ± 0.0 | 0.66 ± 0.1 | 0.75 ± 0.0 |
| *PI_ABS_* | 0.25 ± 0.2 | 0.21 ± 0.1 | 0.18 ± 0.3 | 0.52 ± 0.3 | 0.27 ± 0.1 | 0.75 ± 0.3 |
| *ET_0_/TR_0_* | 0.26 ± 0.1 | 0.24 ± 0.1 | 0.22 ± 0.1 | 0.40 ± 0.1 | 0.35 ± 0.1 | 0.43 ± 0.0 |
| *TR_0_/RC* | 3.11 ± 0.3 | 2.57 ± 0.2 | 3.06 ± 0.2 | 2.73 ± 0.2 | 3.02 ± 0.4 | 2.65 ± 0.1 |
| *ABS/RC* | 4.46 ± 0.7 | 3.72 ± 0.4 | 5.97 ± 1.6 | 3.91 ± 0.5 | 4.54 ± 0.3 | 3.52 ± 0.3 |
| *ET_0_/RC* | 0.77 ± 0.3 | 0.61 ± 0.2 | 0.66 ± 0.4 | 1.08 ± 0.1 | 1.06 ± 0.4 | 1.14 ± 0.1 |
| *DI_0_/RC* | 1.35 ± 0.5 | 1.14 ± 0.2 | 2.91 ± 1.5 | 1.18 ± 0.3 | 1.52 ± 0.2 | 0.88 ± 0.2 |
| *RC/CS* | 126.04 ± 11.9 | 181.77 ± 21.0 | 124.82 ± 26.7 | 183.41 ± 15.0 | 147.07 ± 42.4 | 124.93 ± 16.5 |
| *Dry season and high-nutrient* | | | | | | |
| *F_0_* | 737.00 ± 51.8 | 720.65 ± 65.5 | 737.44 ± 53.8 | 576.71 ± 34.5 | 600.25 ± 95.0 | 460.78 ± 67.7 |
| *F_M_* | 2436.06 ± 302.8 | 2534.60 ± 172.6 | 2148.29 ± 262.1 | 2456.21 ± 100.0 | 2616.50 ± 197.8 | 1990.69 ± 204.2 |
| *F_V_* | 1699.06 ± 339.3 | 1813.95 ± 194.4 | 1410.85 ± 290.5 | 1879.50 ± 97.4 | 2022.75 ± 233.1 | 1529.91 ± 220.5 |
| *F_V_/F_M_* | 0.69 ± 4.5 | 0.71 ± 2.1 | 0.64 ± 9.2 | 0.77 ± 2.0 | 0.77 ± 1.4 | 0.76 ± 2.7 |
| *PI_ABS_* | 0.25 ± 0.2 | 0.25 ± 0.1 | 0.32 ± 0.2 | 0.66 ± 0.3 | 1.28 ± 0.7 | 1.09 ± 0.6 |
| *ET_0_/TR_0_* | 0.26 ± 0.1 | 0.23 ± 0.1 | 0.31 ± 0.1 | 0.38 ± 0.1 | 0.48 ± 0.1 | 0.48 ± 0.1 |
| *TR_0_/RC* | 2.99 ± 0.2 | 2.53 ± 0.1 | 2.51 ± 0.1 | 2.54 ± 0.2 | 2.28 ± 0.2 | 2.59 ± 0.2 |
| *ABS/RC* | 4.35 ± 0.5 | 3.55 ± 0.3 | 3.92 ± 0.5 | 3.32 ± 0.2 | 2.97 ± 0.3 | 3.41 ± 0.4 |
| *ET_0_/RC* | 0.76 ± 0.3 | 0.59 ± 0.2 | 0.76 ± 0.2 | 0.95 ± 0.1 | 1.10 ± 0.2 | 1.23 ± 0.1 |
| *DI_0_/RC* | 1.36 ± 0.4 | 1.03 ± 0.2 | 1.41 ± 0.4 | 0.78 ± 0.1 | 0.69 ± 0.2 | 0.81 ± 0.3 |
| *RC/CS* | 170.71 ± 14.7 | 203.44 ± 13.2 | 190.08 ± 15.1 | 173.94 ± 5.3 | 202.55 ± 27.6 | 135.51 ± 14.6 |
| *Wet season and low-nutrient* | | | | | | |
| *F_0_* | 541.83 ± 77.2 | 623.75 ± 73.7 | 683.17 ± 155.6 | 564.18 ± 64.9 | 525.25 ± 42.2 | 406.17 ± 64.1 |
| *F_M_* | 2006.44 ± 246.6 | 2541.00 ± 257.8 | 1651.78 ± 523.0 | 2359.94 ± 140.9 | 2508.75 ± 312.9 | 2006.78 ± 289.9 |
| *F_V_* | 1464.61 ± 168.1 | 1917.25 ± 319.2 | 968.61 ± 485.5 | 1795.75 ± 192.9 | 1983.50 ± 271.9 | 1600.61 ± 347.4 |
| *F_V_/F_M_* | 0.72 ± 0.1 | 0.75 ± 0.1 | 0.55 ± 0.1 | 0.76 ± 0.0 | 0.79 ± 0.0 | 0.79 ± 0.1 |
| *PI_ABS_* | 0.29 ± 0.2 | 0.47 ± 0.4 | 0.12 ± 0.1 | 0.82 ± 0.5 | 0.86 ± 0.2 | 1.84 ± 1.0 |
| *ET_0_/TR_0_* | 0.27 ± 0.0 | 0.29 ± 0.1 | 0.37 ± 0.1 | 0.41 ± 0.1 | 0.52 ± 0.1 | 0.56 ± 0.1 |
| *TR_0_/RC* | 3.09 ± 0.3 | 2.37 ± 0.2 | 2.80 ± 0.3 | 2.35 ± 0.2 | 2.67 ± 0.3 | 2.32 ± 0.2 |
| *ABS/RC* | 4.31 ± 0.7 | 3.19 ± 0.5 | 5.32 ± 1.5 | 3.11 ± 0.4 | 3.38 ± 0.4 | 2.98 ± 0.5 |
| *ET_0_/RC* | 0.84 ± 0.2 | 0.68 ± 0.2 | 0.58 ± 0.2 | 0.89 ± 0.2 | 1.14 ± 0.1 | 1.15 ± 0.2 |
| *DI_0_/RC* | 1.21 ± 0.5 | 0.81 ± 0.3 | 2.52 ± 1.3 | 0.76 ± 0.2 | 0.71 ± 0.1 | 0.66 ± 0.4 |
| *RC/CS* | 127.70 ± 20.2 | 196.85 ± 16.2 | 135.77 ± 37.9 | 182.57 ± 12.5 | 157.22 ± 23.2 | 136.67 ± 4.4 |
| *Wet season and high-nutrient* | | | | | | |
| *F_0_* | 562.78 ± 52.6 | 622.43 ± 119.9 | 709.23 ± 168.8 | 520.78 ± 59.2 | 571.06 ± 75.5 | 402.28 ± 80.8 |
| *F_M_* | 2567.89 ± 266.7 | 2579.29 ± 208.9 | 2168.11 ± 342.9 | 2411.17 ± 160.7 | 2750.25 ± 228.2 | 2177.06 ± 240.2 |
| *F_V_* | 2005.11 ± 298.5 | 1956.86 ± 302.0 | 1458.88 ± 413.0 | 1890.39 ± 195.2 | 2179.19 ± 180.8 | 1774.78 ± 180.1 |
| *F_V_/F_M_* | 0.78 ± 0.0 | 0.75 ± 0.1 | 0.65 ± 0.1 | 0.78 ± 0.0 | 0.79 ± 0.0 | 0.82 ± 0.0 |
| *PI_ABS_* | 0.67 ± 0.3 | 0.31 ± 0.1 | 0.68 ± 0.6 | 1.15 ± 0.6 | 1.58 ± 0.4 | 2.28 ± 0.5 |
| *ET_0_/TR_0_* | 0.34 ± 0.0 | 0.22 ± 0.1 | 0.37 ± 0.1 | 0.41 ± 0.1 | 0.52 ± 0.1 | 0.56 ± 0.1 |
| *TR_0_/RC* | 2.47 ± 0.2 | 2.50 ± 0.2 | 2.39 ± 0.3 | 2.12± 0.2 | 2.15 ± 0.1 | 2.21 ± 0.1 |
| *ABS/RC* | 3.19 ± 0.3 | 3.33 ± 0.4 | 3.98 ± 1.4 | 2.73 ± 0.3 | 2.71 ± 0.2 | 2.71 ± 0.1 |
| *ET_0_/RC* | 0.84 ± 0.2 | 0.55 ± 0.1 | 0.85 ± 0.2 | 0.87 ± 0.1 | 1.10 ± 0.1 | 1.25 ± 0.1 |
| *DI_0_/RC* | 0.72 ± 0.2 | 0.84 ± 0.3 | 1.59 ± 1.1 | 0.60 ± 0.2 | 0.56 ± 0.1 | 0.50 ± 0.1 |
| *RC/CS* | 176.94 ± 14.1 | 186.44 ± 20.4 | 190.50 ± 25.7 | 191.40 ± 7.9 | 211.12 ± 26.0 | 148.71 ± 31.4 |
