# Supplementary material for: Fertilization and seasonality influence on the photochemical performance of tree legumes in forest plantation for area recovery in the Amazon
Source: PLoS One. 2021 May 21;16(5):e0243118. doi: 10.1371/journal.pone.0243118 (PMC8139463; doi:10.1371/journal.pone.0243118)
Supplement: S2 Table — PIABS, performance index on an absorption basis; DI0/RC, energy dissipation flux. Degrees of freedom for species = 5, and fertilization = 1. * Significance at the 0.05 level; ** Significance at the 0.01 level. (DOCX) [file pone.0243118.s002.docx]

|  |  | **Performance index - *PI*_ABS_** | | **Energy dissipation per active PSII *- DI*_0_/*RC*** | |
| --- | --- | --- | --- | --- | --- |
|  |  | *p* | F | *p* | F |
| ***Factors*** | **Species** | < 0.001 | 39.6 | < 0.001 | 27.1 |
|  | **Fertilization** | < 0.001 | 8.9 | < 0.001 | 20.4 |
|  | **Sp. x Fert.** | 0.02 | 2.8 | < 0.01 | 4.6 |
| ***Repeated measures*** | **Seasonality** | < 0.001 | 35.0 | < 0.01 | 12.5 |
|  | **Season. x Sp.** | < 0.001 | 5.5 | 0.86 | 0.4 |
|  | **Season. x Fert.** | 0.48 | 0.5 | 0.31 | 1.0 |
|  | **Season. x Sp. x Fert.** | 0.67 | 0.64 | 0.30 | 1.2 |
