# Supplementary material for: Fertilization and seasonality influence on the photochemical performance of tree legumes in forest plantation for area recovery in the Amazon
Source: PLoS One. 2021 May 21;16(5):e0243118. doi: 10.1371/journal.pone.0243118 (PMC8139463; doi:10.1371/journal.pone.0243118)
Supplement: Attachment — Submitted filename: Response to Reviewers Jaquetti et al 2021.docx [file pone.0243118.s003.docx]

February 2^nd^, 2021

**To**: Wagner L. Araujo, Ph.D

Academic Editor

PLOS ONE

**Ref. No.:** PONE-D-20-35817

**Title:** Effects of seasonality and fertilization on the photochemical performance of leguminous tree species during Amazonian reforestation

**Rebuttal letter**

Dear Dr. Wagner,

First of all, we are very thankful to excellent editorial work coupled with your, and reviewers as well, critical comments and inputs that were all taken in consideration in production of this revised version. The reviewer has made several valid and useful comments, clearly resulting in an improved version of our manuscript. We have been much more careful and believe that we have addressed all of the reviewers’ comments. Thus, we have corrected all minor mistakes and we have devoted great attention to the major changes suggested by reviewers. Changes were made throughout the manuscript and, in particular, in the discussion section to make the text and ideas more clear. We belief the manuscript has improved substantially. The hypotheses were reformulated to understand the effects of photochemical performance in different species. The figures were merged and presented as suggested. In addition, data is available as a submitted data note of the BMC Research Notes journal. References were double checked and misspellings were revised. Specific arguments are found in red below.

**ANSWERS LIST**

Reviewer #1: The authors present a good work characterizing the use of chlorophyll a fluorescence as a fundamental parameter for identification of trees with potential for reforestation in the Amazon forest. The authors used 6 leguminous tree species and performed a 24-month experiment, analyzing morphophysiological and nutritional characteristics in the dry and rainy seasons, in addition to treatments with high and low nutritional availability. The Abstract section needs to be rewritten to make the idea of the manuscript more evident, **for instance, talking about which species were used (and why?) and about the aim of testing chlorophyll a fluorescence as a technique for selection and/or classification of species** with potential for reforestation. The authors also need to reorganize the **Introduction and write about which species were used and why.** The Methodology is well presented, but crucial **information about the location of the experiment and climatology is lacking**. IMHO, **most of the results at supplementary data should be presented in the body of the manuscript, in Results section** but not only, because as currently form of the manuscript it is **hard to interpret and understand the data**. Furthermore, it is not clear which parameters were used to define whether data is supplementary or necessary for the manuscript. The Discussion is **exceedingly difficult to follow regarding the species used** in the experiment because they were not presented properly at the beginning of the Manuscript. **Besides that, the results (figures and tables) need to be cited and highlighted in the Discussion section. The layout and presentation of Figures, Tables and Supplementary data need to be changed to improve the understanding of the manuscript.**

**Authors' response:** We are pleased to receive such comments and truly appreciate the support to our work. Briefly, all issues raised were prompt accepted. Abstract was revised in intent to evidence the use of chlorophyll fluorescence technique and to give ecological and silvicultural basic information of the species. The introduction was revised and more emphasis was given to species ecological groups and N-fixing species. The first and third hypotheses were reformulated to address how chlorophyll fluorescence can be used to screen stress and performance responses of potential species for restoration.

More detail was given about the location and and climate of the region, however the precipitation and soil characteristics values are presented in the data note manuscript submitted to BMC Research Notes and data files available from: <https://doi.org/10.6084/m9.figshare.12869960.v3>. Efforts were made to simplify the interpretation and presentation of data results. Supplementary figures were merged and included in the results and discussion sections. Supplementary Tables 1 and 2 were included in the main text.

The discussion section was restructured to address: 1. Specific responses on chlorophyll fluorescence main variables under high-light conditions of degraded areas; 2. The effects of seasonality and fertilization treatments on photochemical performance; and 3. How fluorescence variables can be used as proxy for photosynthesis and growth determination. As wisely suggested concise conclusions and a brief summary were used in each topic of the discussion section with significant improve to the meaning of the text.

Point-by-point revision:
Effects of seasonality, fertilization and species on the chlorophyll a fluorescence as related with photosynthesis and leguminous tree growth during Amazonian forest restoration
The title needs to be improved, for now it is too long and messy.

**Authors' response:** The title was shortened with the intention of clarifying the understanding. The photochemical element was added to improve readers attention.

Abstract
L3-5: Remove “General and specific” and Rewrite. Merge these sentences. If these “conditions have been studied” what we know until now?

**Authors' response:** Corrections were made as appropriate suggested.

L8: Please rewrite this sentence.

**Authors' response:** Corrections were made as appropriate suggested.

L9: Remove “Additionally,”

**Authors' response:** Correction was made as suggested.

L14: What type of “performance index”?

**Authors' response:** Corrections were made for performance index on an absorption basis (*PI*_ABS_) as appropriate suggested.

L16: Change to “High”

**Authors' response:** Correction was made as suggested.

L17-19: Why?

**Authors' response:** The sentence was reformulated to evidence the coordinated responses among performance index and other traits as follows “N-fixers showed increased performance index as correlated to electron transport, zinc and nitrogen foliar concentrations.”

L21-23: If the work was (at least partially) a proof of concept of these "cost-effective technique" this information should be described in the beginning of Abstract.

**Authors' response:** No data comparing costs of diameter and biomass growth, chlorophyll fluorescence and photosynthesis data collection was performed. For instance we belief that chlorophyll fluorescence is less expensive than biomass and photosynthesis determination. Still as no costs data are available we remove the cost aspect from the text.

L24: Remove “greatly”.

**Authors' response:** Correction was made as suggested.

Introduction

L33-34: How these N_2_-fixing tree species enhance light uptake? What the links between soil nutrition and light assimilation? Pigment production? Explore this topic.

**Authors' response:** The topic was better explored in the discussion section through lines 333-336.

L38: “Various”? What effects? Describe these effects on photochemical components.

**Authors' response:** Corrections were made as appropriate suggested.

L39: What type of “performance index”?

**Authors' response:** Corrections were made as appropriate suggested.

L40: Which “tested species”? Trees? Crops? Native species? Model species?

**Authors' response:** Corrections were made as appropriate suggested and information about the species was included.

L44-45: Reported in which species?

**Authors' response:** Corrections were made as appropriate suggested and information about the species was included.

L61-62: Why these species are important?

**Authors' response:** The ecological importance of these species was discussed in the proper section the term was excluded as appropriate suggested.

L63: Describe and present these “few studies”.

**Authors' response:** Studies were properly cited and briefly described in the introduction, and better presented in the discussion section.
L65: The authors need to talk more about "Leguminous trees" in the Introduction, which species were used in this work?

**Authors' response:** More detail on ecological aspects of the species used and N-fixing group is now described in the second paragraph of introduction.

L66: Explore more the “Evolutionary N_2_-fixing species” and remove “will”.

**Authors' response:** The changes were made as suggested, and the topic of evolving nodulation in legume was presented with the species in the beginning of the introduction.

Materials and methods
L71-73: Add some reference about climate characteristics, for instance: Alvares et al. 2013 (<https://doi.org/10.1127/0941-2948/2013/0507>).

**Authors' response:** Corrections were made as appropriate suggested. References about soil and climate characteristics were added.

L73-74: Remove “Without delving into geopolitical and scientific discussion”.

**Authors' response:** The text was removed as appropriate suggested.

L74-75: Add references about “Large- and small-scale degradation” in Amazon basin, for instance:

https://doi.org/10.1038/s41893-020-0492-y; https://doi.org/10.1073/pnas.1905315116; https://doi.org/10.1038/srep41489; https://doi.org/10.1111/nyas.14364; https://doi.org/10.1126/sciadv.aba2949; https://doi.org/10.1038/nclimate3238; https://doi.org/10.1038/s41467-017-02771-y

**Authors' response:** References about deforestation in Amazonia were added as suggested.

L77: “Located two hours by car from Manaus”? Please use distance in Kilometers and add the geographic coordinates.

**Authors' response:** Information about location of the experimental area was added.

L80: What means “good physical characteristics of soil”? Which one traits and what values?

**Authors' response:** References were presented and changes to the text were made as appropriate recommended.

L82: Where “Natural regeneration was not observed”? Describe better.

**Authors' response:** Corrections were made as appropriate recommended.

L87: Change “season” to “seasons”.

**Authors' response:** Corrections were made as recommended.

L105-107: This is Results and/or Discussion.

**Authors' response:** Corrections were made as appropriate recommended. The paragraph was rephrased and presented with the importance of N-fixing species in the introduction section.

L113-115: This information needs to be easily accessed. Climatological data should be presented in the manuscript. Add this information at least as Supplemental Information.

**Authors' response:** A paper with information on soil and precipitation data on the experimental area has been submitted to BMC Research Notes and is under revision. The data reference was included in the references section. Data files and raw dataset are freely available at:

<https://doi.org/10.6084/m9.figshare.13130279.v3>

<https://doi.org/10.6084/m9.figshare.12869960.v3>
Changes to the text were made as appropriate recommended.

L119: Why “east side”?

**Authors' response:** The leaves from the east side are selected because of methods standardization since is the part of the crown fully exposed to sunlight in the first morning hours.

L122: Light curve

**Authors' response:** Corrections were made in the text to improve the intended meaning.

L132: Demonstrate in the Methods how this “Performance Index” was calculated.

**Authors' response:** The equation used to calculate Performance index was presented in the Methods.

L133: “Table S2” should be present in the manuscript body, not as supplementary information.

**Authors' response:** The referred table was included in the body of the manuscript as Table 1.

L153: “S1 Table” is Results.
**Authors' response:** The referred table was included in the body of the manuscript and presented in the results section as Table 2.

Results
L166: “S1 Table” should not be supplementary data and it must be presented in the body of the manuscript.

**Authors' response:** The referred table was included in the body of the manuscript and presented in the results section as Table 2.

L168: S2 Table should not be supplementary data and it must be presented in the body of the manuscript.
**Authors' response:** Corrections were made as recommended.

L172: “S1 Table and S1 Fig” should not be supplementary data and it must be presented in the body of the manuscript.

**Authors' response:** The referred table was included in the body of the manuscript as Table 1.S1 Fig was merged with Fig 2.

L183: “S1 Table” should not be supplementary data and it must be presented in the body of the manuscript.

**Authors' response:** The referred table was included in the body of the manuscript and presented in the results.

L192: “S3 Table”, it is OK this table be present in supplementary information.

L203: “S4 Table”, it is OK this table be present in supplementary information.

L225: “Table 1” should be “Table 3”.

**Authors' response:** Corrections were made as recommended.

Discussion
L241: It is important that the Discussion starts with a sentence from the authors, a summary of all results and initial conclusion.

**Authors' response:** As wisely suggested a concise conclusion and a brief summary were used in each topic of the discussion section improving the flow of the discussion.

L242: Add “availability” between “nutrient” and “effects”.

**Authors' response:** Corrections were made as recommended.

L261-264: How this novel finding is related with your main hypothesis?

**Authors' response:** The hypothesis was reformulated to address how growth and photosynthesis are explained by fluorescence variables.

L265-277: How these information of other authors is related with your findings in this work? Please, merge these paragraphs and add a partial conclusion.

**Authors' response:** The relation between our and previous findings was better presented in the discussion section lines 377-383.

L286: Please, add a conclusion for this topic.

**Authors' response:** Corrections were made as recommended.

L292-295: Invert the order of these sentences, it will facilitate reading avoiding misinterpretation.

**Authors' response:** Corrections were made as recommended.

L301: Please, add a conclusion for these paragraph and topic.

**Authors' response:** Corrections were made as recommended.

L303: Remove the commas.

**Authors' response:** Corrections were made as recommended.

L304: Add a dot after “energy”.

**Authors' response:** Corrections were made as recommended.

L310: Please, add a conclusion for this paragraph.

**Authors' response:** Corrections were made as recommended.

L318: Please, add a conclusion for these paragraph and topic.

**Authors' response:** Corrections were made as recommended.

L319: This topic needs to be presented in the Results section and then discussed here.

**Authors' response:** Corrections were made as recommended.

L324: “S3 Fig” should not be supplementary data and it must be presented in the body of the manuscript.

**Authors' response:** Corrections were made as recommended.

Resp. The referred OJIP curves figure was included in the body of the manuscript as Figure 5 and presented in the Results section.

**Authors' response:** Corrections were made as recommended.

Conclusions
Good conclusion and in agreement with Results.

Figures
Fig 1 - OK
Fig 2 – merge Fig 2 (high nutrition PCA) with Fig S1 (low nutrition PCA), and present as A and B, respectively.

**Authors' response:** Figures were merged as recommended.

Fig 3 – merge Fig 3 (wet season PCA) with Fig S2 (dry season PCA), and present as A and B, respectively.

**Authors' response:** Figures were merged as recommended.

Fig 4 – merge Fig 4 with Fig 5, and present as A and B, respectively.

**Authors' response:** Figures were merged as recommended.

Fig 5 – use Fig S3, present the OJIP curves in the Results section.

**Authors' response:** The referred OJIP curves figure was included in the body of the manuscript as Figure 5 and presented in the Results section.

Fig 6 – OK

Tables OK
Table 1 – use the S2 Table (firstly presented in the Methods section)

**Authors' response:** Corrections were made as recommended.

Table 2 – use the S1 Table OK
Table 3 – use the Table 1 OK

Supplementary information OK
- Climatological data OK
- S3 Table (rename to S1 Table)

**Authors' response:** S3 Table was renamed to S1.

- S4 Table (rename to S2 Table)

**Authors' response:** S4 Table was renamed to S2.

- Raw data OK

Once again, we are very thankful for your editorial work and the opportunity of submitting a much polished work and to improve our study, if you have any questions or new suggestions, please let us know and we shall be satisfy to include them.

Thank you for considering our manuscript.

Roberto Kirmayr Jaquetti Ph.D.

National Institute for Amazonian Research (MCTI-INPA)

Laboratory of Plant Physiology and Biochemistry / PO Box 2223

Manaus-AM, Brazil
