# Supplementary material for: Fertilization and seasonality influence on the photochemical performance of tree legumes in forest plantation for area recovery in the Amazon
Source: PLoS One. 2021 May 21;16(5):e0243118. doi: 10.1371/journal.pone.0243118 (PMC8139463; doi:10.1371/journal.pone.0243118)
Supplement: Attachment — Submitted filename: Response to reviewers PONE-D-20-35817.docx [file pone.0243118.s004.docx]

April 2^nd^, 2021

Point-by-point revision:
**Responses to Reviewer #1:**

The authors improved the manuscript, and the message is much clearer now. However, the work still needs to be changed to be fully accepted. Below are my new comments.

**Authors' response:** The authors are much appreciated with your revisions and comments. All suggestions were incorporated with much improve to the manuscript significantly.

*Clitoria*, *Inga* and *Acacia* are genus, not species. The same for *Cenostigma*, *Senna* and *Dipteryx*. Please correct this in all manuscript. If authors cannot confirm the species used in the experiments, add "spp." after the genus name in all text, figures, and tables. In the previous version of the manuscript, the way that the species name was presented in the figure 1 captions was correct, now the authors only present the name of the genus.

**Authors' response:** The name of species were changed as previously presented. *Acacia* was not confirmed to species level, therefore spp. was used.

Moreover, please standardize the use of "N" or "nitrogen" in the text. Remember that once the chemical element symbol is displayed, there is no need to write its name again.

**Authors' response:** As suggested the abbreviation of chemical element symbols were standardize and revised in the main text.
